# SHAPE directed RNA folding

**DOI:** 10.1093/bioinformatics/btv523

**Published:** 2015-09-09

**Authors:** Ronny Lorenz, Dominik Luntzer, Ivo L. Hofacker, Peter F. Stadler, Michael T. Wolfinger

**Affiliations:** ^1^Institute for Theoretical Chemistry, University of Vienna, Vienna, Austria,; ^2^Bioinformatics Group, Department of Computer Science, and Interdisciplinary Center of Bioinformatics, University of Leipzig, 04109 Leipzig, Germany,; ^3^Research Group BCB, Faculty of Computer Science, University of Vienna, Vienna, Austria,; ^4^Center for Integrative Bioinformatics Vienna (CIBIV) and Department of Biochemistry and Molecular Cell Biology, Max F. Perutz Laboratories, Vienna, Austria

## Abstract

**Summary**: Chemical mapping experiments allow for nucleotide resolution assessment of RNA structure. We demonstrate that different strategies of integrating probing data with thermodynamics-based RNA secondary structure prediction algorithms can be implemented by means of soft constraints. This amounts to incorporating suitable pseudo-energies into the standard energy model for RNA secondary structures. As a showcase application for this new feature of the ViennaRNA Package we compare three distinct, previously published strategies to utilize SHAPE reactivities for structure prediction. The new tool is benchmarked on a set of RNAs with known reference structure.

**Availability and implementation**: The capability for SHAPE directed RNA folding is part of the upcoming release of the ViennaRNA Package 2.2, for which a preliminary release is already freely available at http://www.tbi.univie.ac.at/RNA.

**Contact**: michael.wolfinger@univie.ac.at

**Supplementary information:** Supplementary data are available at *Bioinformatics* online.

## 1 Introduction

Beyond its role as information carrier from genome to proteome, RNA is a key player in genome regulation and contributes to a wide variety of cellular tasks. The spatial structure of RNA plays an important role in this context because it critically influences the interaction of RNAs with proteins and with nucleic acids. Knowledge of RNA structure is therefore crucial for understanding various biological processes. Chemical and enzymatic probing methods provide information concerning the flexibility and accessibility at nucleotide resolution. They are based on the observation that RNA can be selectively modified by small organic molecules, metal ions or RNAse enzymes, resulting in formation of an adduct between the RNA and the small compound or RNA cleavage. Subsequent primer extension mediated by RT enzymes typically terminates at the modified sites. The resulting cDNA fragments thus inform directly on the RNA structure by identifying, depending on the particular reagent, paired or unpaired sequence positions. For a recent overview of such (high-throughput) probing methods we refer to Mortimer *et* *al.* (2014).

As chemical probing is becoming a frequently used technology to determining RNA structure experimentally, there is increasing demand for efficient and accurate computational methods to incorporate probing data into secondary structure prediction tools. Efficient dynamic programming algorithms, as implemented in the ViennaRNA Package (Lorenz *et* *al.*, 2011), typically yield excellent prediction results for short sequences, but accuracy decreases to between 40 and 70% for long RNA sequences. This discrepancy is mainly caused by imperfect thermodynamic parameters and the inherent limitations of the secondary structure model, such as tertiary interactions, pseudoknots, ligand binding or kinetics traps. To alleviate the gap in available computational tools we have extended the ViennaRNA Package by a flexible framework to incorporate all those *soft constraints* that are compatible with the RNA folding grammar; here we use this to handle position-wise data as they arise from chemical probing experiments.

## 2 Methods

In contrast to *hard constraints* (Mathews *et* *al.*, 2004), which restrict the folding space on the level of the generating function, *soft constraints* leave the structure ensemble intact. They rather guide the folding process by adding position-, or motif-specific pseudo-energy contributions to the free energy contributions of certain loop motifs. This amounts to a distortion of the equilibrium ensemble of structure in favour of those that are consistent with experimental data. Mismatching motifs are penalized by positive contributions, while structure patterns where prediction and experiment agree with each other receive a ‘bonus’ in form of a negative pseudo-energy. Bonus energies are an old idea in RNA folding algorithms (see Supplementary Material).

Current methods for guided secondary structure prediction by means of soft constraints mainly focus on the incorporation of SHAPE reactivity data. For that purpose, three algorithms are available that aim to transform normalized SHAPE reactivity data into meaningful pseudo-energy terms. The first method published uses a simple linear ansatz to derive pseudo-energies for individual nucleotides that take part in a stacked helix conformation (Deigan *et* *al.*, 2009). All remaining structural conformations are not modified in this model. A more consistent model that considers pseudo-energy guided free energy modifications in all loop types was introduced by Zarringhalam *et* *al.* (2012). Here, the authors first convert the provided SHAPE reactivity data for each nucleotide into a probability to be unpaired. Subsequently, the resulting probabilities are used to derive two nucleotide-wise pseudo-energy weights, one for contexts where the nucleotide is considerd unpaired, and the other for situations where it is involved in a base pair. A third, distinct approach on incorporating SHAPE reactivity data to guide secondary structure prediction was suggested by Washietl *et* *al.* (2012). Here, the authors phrase the choice of the bonus energies as an optimization problem that aims to find a perturbation vector of pseudo-energies that minimizes the discrepancy between the observed and predicted probabilities to see particular nucleotides unpaired. At the same time, the perturbation should be as small as possible. The tradeoff between the two goals is naturally defined by the relative uncertainties inherent in the SHAPE measurements and the energy model, respectively. A detailed description of the three conversion methods is given in the Supplementary Material.

### 2.1 Implementation

All three methods outlined earlier have been implemented into the ViennaRNA Package, and are available via the API of the ViennaRNA Library and the command line interface of RNAfold. The required changes to the folding recursions and technical details of handling both hard and soft constraints in ViennaRNA will be described elsewhere in full detail. The key feature for our purposes is the consistent incorporation of a user defined position dependent energy contribution for each nucleotide that remains unpaired. The novel standalone tool RNApvmin dynamically estimates a vector of pseudo-energies that minimize model adjustments and discrepancies between observed and predicted pairing probabilities. The resulting perturbation vector can then be used to guide structure prediction with RNAfold. By accepting either SHAPE reactivity data, probabilities to be unpaired, or bonus energies directly, RNAfold allows to incorporate alternative ways of computing bonus energies, e.g. along the lines of Eddy (2014), or the application to other types of probing data. The novel soft constraint feature introduces a variety of parameters which need to be chosen carefully. We refer to the Supplementary material for a detailed summary of their default values. Guided structure prediction has also been included into the ViennaRNA Websuite (Gruber *et* *al.*, 2008), available at http://rna.tbi.univie.ac.at.

## 3 Results

**Fig. 1.**
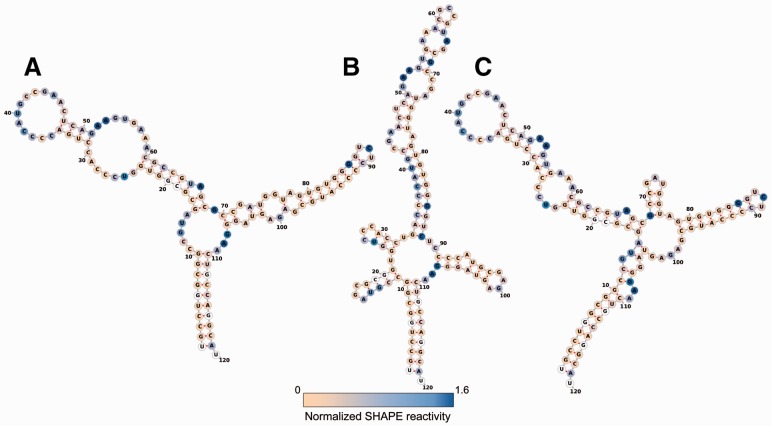
Secondary structure prediction of *Escherichia coli* 5S rRNA from our benchmark data set. (**A)** Structure reference, (**B)** prediction by RNAfold with default parameters and (**C)** prediction by RNAfold with guiding pseudo-energies obtained from SHAPE reactivity data using RNApvmin. Structure plots created using the forna Web server (Kerpedjiev *et al.*, 2015). White nucleotides correspond to missing SHAPE reactivity data

We applied the methods to a benchmark set with known reference structures (Hajdin *et* *al.*, 2013). This test set contains 24 triples of sequences, their corresponding SHAPE data, and reference structures, either derived from X-ray crystallography experiments or predicted by comparative sequence analysis. The use of SHAPE data driven soft constraints leads to improved prediction results for many RNAs. This is clearly visible in the predictions for our benchmark data set (see Fig. 1, and Supplementary Material). However, for some of the RNAs within our benchmark data the additional pseudo-energy terms impair prediction results. This may be due to two factors. First, experimental data always comes with a certain inaccuracy. Second, the underlying energy model excludes pseudoknotted structures, which are present in approximately half of the benchmarked RNAs. Additionally, pseudoknot interactions are reflected in the SHAPE data itself.

Incorporation of probing data not only affects the minimum free energy structure, but also the entire ensemble of structures. Consequently, the predicted pairing probabilities are shifted towards the observed reactivity pattern. However, the effect is less distinct in the model of Washietl *et* *al.* (2012) (see Supplementary Fig. S11). While Deigan’s method has the best average performance on our data, neither approach consistently outperforms the others.

In addition to the benchmark data, we use an artificially designed theophylline sensing riboswitch to compare the three SHAPE conversion methods with a prediction that directly includes ligand binding free energy of the aptamer (see Supplementary Material S5).

## Supplementary Material

Supplementary Data
