# Supplementary material for: SHAPE directed RNA folding
Source: Bioinformatics. 2015 Sep 9;32(1):145–7. doi: 10.1093/bioinformatics/btv523 (PMC4681990; doi:10.1093/bioinformatics/btv523)
Supplement: Supplementary Data [file supp_32_1_145__index.html]

SHAPE directed RNA folding — SHAPE directed RNA folding — Supplementary Data 

# SHAPE directed RNA folding

## Supplementary Data

files

- Supplementary Data - pdf file
