# Supplementary material for: SHAPE directed RNA folding
Source: Bioinformatics. 2015 Sep 9;32(1):145–7. doi: 10.1093/bioinformatics/btv523 (PMC4681990; doi:10.1093/bioinformatics/btv523)
Supplement: Supplementary Data [file supp_btv523_supplement_updated.pdf]

SHAPE directed RNA folding
# - Supplementary Material -

Ronny Lorenz [2,1]*, Dominik Luntzer [1]*, Ivo L. Hofacker [1,3,4]
Peter F. Stadler [1,2,4,7,8,9], and Michael T. Wolfinger [1,5,6,†]

[1]Institute for Theoretical Chemistry, University of Vienna, Währingerstraße 17/3, A-1090 Vienna, Austria.
[2]Department of Bioinformatics, University of Leipzig, Härtelstraße, 16-18, 04109 Leipzig, Germany.
[3]Research group Bioinformatics and Computational Biology, Faculty of Computer Science, University of Vienna, Währingerstraße 29, 1090 Vienna, Austria.
[4]Center for RNA in Technology and Health, University of Copenhagen, Grønnegårdsvej 3, Frederiksberg C, Denmark.
[5]Center for Integrative Bioinformatics Vienna (CIBIV), Max F. Perutz Laboratories, University of Vienna, Medical University of Vienna, Dr. Bohr-Gasse 9, 1030 Vienna, Austria.
[6]Department of Biochemistry and Molecular Cell Biology, Max F. Perutz Laboratories, University of Vienna, Dr. Bohr-Gasse 9, 1030 Vienna, Austria.
[7]Max Planck Institute for Mathematics in the Sciences, Inselstraße 22, D-04103 Leipzig, Germany.
[8]Fraunhofer Institute for Cell Therapy and Immunology, Perlickstraße 1, D-04103 Leipzig, Germany.
[9]Santa Fe Institute, 1399 Hyde Park Road, Santa Fe NM 87501.

1 Availability

The complete set of benchmark data is available for downloaded at `http://github.com/dluntzer/shapebenchmark`. These data may be of use for future comparisons against novel or updates approaches.

2 Related Work

Soft constraints are naturally implemented in folding algorithms using bonus energies. Since the size limit on Application Notes makes it impossible to include a proper review of the pertinent literature in the main text, we briefly summarize the relevant literature in this section of the Supplement.

The idea of guiding RNA folding by adding pseudo-energies to specific terms in the recursion goes back all the way to early implementations of hard constraints for excluding or forcing base pairs in early versions of `mfold` Zuker *et al.* (1991) and the `ViennaRNA Package` Hofacker *et al.* (1994). Although this particular form of pseudo-energies has been replaced by hard constraints on the recursions in more recent implementations of RNA folding algorithms, see e.g. Mathews *et al.* (2004), the concept of pseudo-energies terms has been persistent.

*These authors contributed equally to this work
†To whom correspondence should be addressed

For a while, exceptions to general energy rules were handled as position-specific bonus energies on top of a standard energy model (e.g. in the Turner 1999 model, Mathews *et al.* (1999). Probably the best known example are the bonus energies for extra-stable tetraloops.

Bonus energies, in this case derived from sequence covariation, were also used to guide consensus folding in `RNAalifold` Hofacker *et al.* (2002); Bernhart *et al.* (2008). Similarly, `TurboFold` Harmanci *et al.* (2011) makes use pseudo-energies rewarding conservation of local structure.

Soft constraints have gained a more focussed interest recently in the context of analyzing chemical and enzymatic probing experiments. In `RNAstructure` Deigan *et al.* (2009); Zarringhalam *et al.* (2012); Hajdin *et al.* (2013), the first application of this type, SHAPE reactivities are converted to position-specific stabilizing energies for unpaired bases. The same idea, albeit with different models of bonus energies, has been used successfully also for other types of chemical probing e.g. using DMS Cordero *et al.* (2012) and to enymatic probing (PARS Kertesz *et al.* (2010); Wan *et al.* (2014)). A variation on this theme has been proposed in Washietl *et al.* (2012). Instead of computing the bonus energies directly from the reactivities, they are determined here as the solution of an optimization problem that tries to balance the experimental signal and the thermodynamic folding model. Full probabilistic models for the task are advocated in Eddy (2014).

3 Metrics

3.1 Minimum free energy structure

In order to rate the quality of RNA secondary structure prediction results in terms of prediction accuracy, the predicted minimum free energy structures are usually compared to known reference secondary structures. Suboptimal folds, yielding a free energy within a certain range from the minimum free energy structure, may also contain structures with similar or even better quality than the MFE structure. However, there is no way to rate the quality of suboptimal folds when the reference structure is unknown, which is usually the case. As a result, suboptimal folds can not be used in a meanigful to evaluate the quality of a secondary structure prediction algorithm.

The correctness of the minimum free energy structure prediction is evaluated by comparing the predicted base pairs against the base pairs determined from the reference structure. In order to measure the quality, two parameters are most commonly used to describe the amount of correct and wrong base pair predictions: The *Sensitivity* represents the percentage of base pairs in the reference structure, which are also found in the prediction. However, many RNA secondary structure prediction algorithms tend to predict additional pairs, which can not be verified with experimental methods. As a result the *Positive predictive value* (PPV), that is, the fraction of predicted base pairs that are also present in the reference structure, is used to measure the amount of false positives.

$$\text{Sensitivity} = \frac{\text{Number of correctly predicted base pairs}}{\text{Number of base pairs in the reference structure}}$$
$$\text{Positive Predictive Value} = \frac{\text{Number of correctly predicted base pairs}}{\text{Number of predicted base pairs}} \tag{1}$$

3.2 Partition function

In contrast to the MFE structure, the partition function approach is used to model the whole ensemble of structures instead of predicting just one or a few promising secondary structure candidates. Several different parameters can be used to describe the agreement of the predicted ensemble with the known reference structure.

The *Pairing Probability Score* is defined as the arithmetic mean of the predicted pairing probabilities p_{ij} of all pairs contributing to the reference structure S and shows the agreement of the pairing probability matrix derived from the ensemble of all possible structures with one single reference structure:

$$\text{Pairing Probability Score} = \frac{1}{|S|} \sum_{(i,j)\in S} p_{ij} \tag{2}$$

The *Ensemble Diversity* $\langle d \rangle$ shows the mean distance between predicted pairs, which can be obtained from the predicted pairing probabilities. Since the algorithms for incorporating experimental data tend to favor motifs that are in agreement with the observed experimental data, while penalizing disagreeing motifs, the *Ensemble Diversity* is used to illustrate to which extent the shift towards the experimental data influences the variability of the secondary structures represented by the ensemble. The *Ensemble Diversity* of a thermodynamic based prediction depends on the energy model and its parameters. Since the incorporation of additional experimental information is usually done by adding additional constraints, a decrease in the *Ensemble Diversity* in contrast to the thermodynamic based prediction is expected. However, a large decrease in ensemble diversity indicates a major shift towards probing data, thus rendering equilibrium properties such as base pair probablilities uninformative. Since the amount of possible pairs raises with growing sequence lengths, the *Ensemble Diversity* is normalized through division by the length n of the RNA to ensure comparability between RNAs of different size.

$$\langle d \rangle = \sum_{i,j\in S} p_{ij}(1 - p_{ij}) \tag{3}$$

The *Ensemble Distance* is the L^1-distance between the predicted ensemble and the reference structure:

$$\langle d(S) \rangle = \sum_{i,j\in S} (1 - p_{ij}) + \sum_{i,j\notin S} p_{ij} \tag{4}$$

It measures the agreement of the predicted ensemble with the accepted target structure quantitatively. In contrast to the *Pairing Probability Score*, which

focuses on the predicted pairing probabilities for base pairs present in the target structure, here the probabilities for all possible basepairs are taken into account. Since the incorporation of experimental data into prediction algorithms tends to prefer structures being in accordance with the determined structural features, the ensemble distance can be used to quantify the expected shift of the whole ensemble towards the reference structure.

It should be noted that all of the ensemble properties mentioned above cannot be used by themselves as measures of prediction quality. They are useful, however, to help interpret the predicted ensembles in comparison to each other. In particular, they provide insights how strongly the ensemble of structures is distorted by the constraints. Furthermore, the extent of the resulting measures generally grows linearly with sequence length, and should therefore be normalized by the sequence length n. This ensures compareability of predictions for RNAs of different length.

4 Conversion of SHAPE reactivities into pseudo free energy terms

In this section we briefly discuss the implementation of three different version for how to include chemical probing data, in particular SHAPE reactivities, into the `ViennaRNA Package`.

The Deigan *et al.* (2009) method was the first approach to incorporate SHAPE data to direct RNA folding. It uses the following simple heuristic model for the pseudo-energies

$$\Delta\ G_{\text{SHAPE}}(i) = m \ln(\text{SHAPE reactivity}(i) + 1) + b.$$

as a function of the measured SHAPE reactivity values for a given nucleotide i. This energy contributes to a stacked pair (Deigan *et al.*, 2009). A positive slope m penalizes high reactivities in paired regions, while a negative intercept b results in a confirmatory "bonus" free energy for correctly predicted base pairs. Since the energy evaluation of a base pair stack involves two pairs, the pseudo energies are added for all four contributing nucleotides. Consequently, the energy term is applied twice for pairs inside a helix and only once for pairs adjacent to other structures. For all other loop types the energy model remains unchanged even when the experimental data highly disagrees with a certain motif.

A somewhat more principled model considers nucleotide-wise experimental data in all loop energy evaluations (Zarringhalam *et al.*, 2012). First, the observed SHAPE reactivity of nucleotide i is converted into the probability q_i that position i is unpaired by means of a non-linear map. Then pseudo-energies of the form

$$\Delta\ G_{\text{SHAPE}}(x, i) = \beta\ |x_i - q_i|\,,$$

are computed, where $x_i = 0$ if position i is unpaired and $x_i = 1$ if i is paired in a given secondary structure. The parameter β serves as scaling factor. The magnitude of discrepancy between prediction and experimental observation is represented by $|x_i - q_i|$.

These two methods incorporate pseudo-energies even when the observed data are consisted with an unaided secondary structure prediction. In an attempt

to avoid pseudoenergy contribution to positions that are already predicted correctly based by the thermodynamic model, Washietl *et al.* (2012) suggested to phrase the choice of the bonus energies as an optimization problem aiming to find a perturbation pseudo-energy vector $\vec{\epsilon}$. The perturbation is chosen in such a away that the discrepancy between the observed and predicted probabilities to see position i unpaired, respectively, is minimized. At the same time, the perturbation should be as small as possible. The tradeoff between the two goals is naturally defined by the relative uncertainties inherent in the SHAPE measurements and the energy model, respectively. An appropriate error perturbation vector thus satisfies

$$F(\vec{\epsilon}) = \sum_{\mu} \frac{\epsilon_{\mu}^2}{\tau^2} + \sum_{i=1}^{n} \frac{(p_i(\vec{\epsilon}) - q_i)^2}{\sigma^2} \to \min .$$

Here, ϵ_{μ} is the perturbation energy for a certain structural element μ and the variances τ_{μ}^2 and σ_i^2 serve as weighting factors for the relative influence of the structure predicted from the standard energy model compared to the experimental data.

In this setting, the energy model is only adjusted when necessary. If the thermodynamic model already yields a perfect prediction, the resulting perturbation vector vanishes and the folding recursions remain unbiased. Otherwise the perturbation vector is used to guide the folding process by adding a pseudo-energy ϵ_i whenever nucleotide i appears unpaired in the folding recursions.

Since the pairing probabilities are derived from the whole ensemble of secondary structures, the algorithm of Washietl *et al.* (2012) tends to decrease structural diversity only slightly, which makes it applicable to RNAs with several distinct low free energy structures. Furthermore, the inferred perturbation energies identify sequence positions that require major adjustments of the energy model to conform with the experimental data. High perturbation energies for just a few nucleotides are therefore indicative of posttranscriptional modifications or intermolecular interactions that are not explicitly handled by the energy model. A major drawback of this approach is its asymptotic time complexity of $O(n^4)$, which renders it very expensive for long sequences. This can be alleviated by a sampling strategy for estimating the gradient of the error functional F and provides a viable alternative to the exact numeric solution that reduces the time complexity to $O(n^3)$ again.

5 Benchmark Data

The test set created by Hajdin *et al.* (2013) was used for benchmarking the accuracy of secondary structure predictions including SHAPE data (`http://www.chem.unc.edu/rna/data-files/ShapeKnots_DATA.zip`). It consists of 24 sequences with their corresponding SHAPE data sets and reference structures, which are required to score the prediction results. The reference structures were derived from X-ray crystallography experiments, or predicted by comparative sequence analysis. As shown in Figure 1, the benchmark shows a high diversity regarding the length and prediction performance of the involved RNAs.

The test set described above has been designed for benchmarking an $O(n^6)$ algorithm that predicts secondary structures containing pseudoknots. As a result the longest RNA sequences in the test set have a length of just about 500

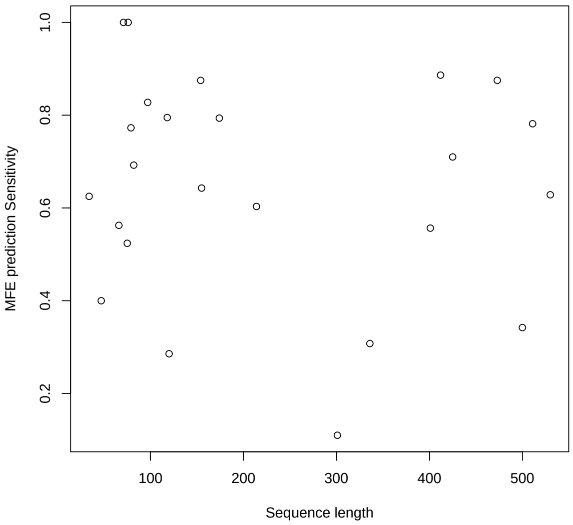

Figure 1: Length and MFE prediction *Sensitivity* of RNAs used for benchmarking.

nucleotides. The benchmark set also contains target structures without pseudoknots. The `ViennaRNA Package` does not support pseudoknot prediction. Of course, this prohibits perfect predictions for benchmark structures containing pseudoknots. On the up side, the computational cost is only $O(n^3)$, thus allowing much larger RNAs, including in particular SSU and LSU ribosomal RNAs, to be processed.

All three methods compared here depend on a set of carefully adjusted parameters. For the methods of Deigan *et al.* (2009), and Zarringhalam *et al.* (2012) we use the latest published default parameters of $m = 1.8$, $b = -0.6$, and $\beta = 0.8$, respectively. Since there are no default parameters available for the stochastic version of the Washietl *et al.* (2012) method, we performed an exhaustive evaluation of its parameter space, see Figure 2. From this analysis, we selected new default parameters which will be described below. The result of the corresponding leave-one-out cross validation is shown in Figure 3. It should be noted, that the above mentioned parameters for the Deigan *et al.* (2009) method were already trained on a majority of the 24 reference RNAs from our benchmark set in another study Hajdin *et al.* (2013).

Our implementation of the method by Washietl *et al.* (2012) in the program `RNApvmin` defaults to an estimation of the gradient by drawing 1000 sample structures from the Boltzmann ensemble. This not only considerably speeds up the optimization routines, but also enables their application to rugged landscapes where an exact gradient approach could easily trap the optimization process in a local minimum. Based on the data from an exhaustive parameter space evaluation, we selected the following default combination for this approach: $\tau/\sigma = 1.0$, minimizer tolerance $\epsilon_m = 0.001$, initial step size of the minimizer method $s_m = 0.01$.

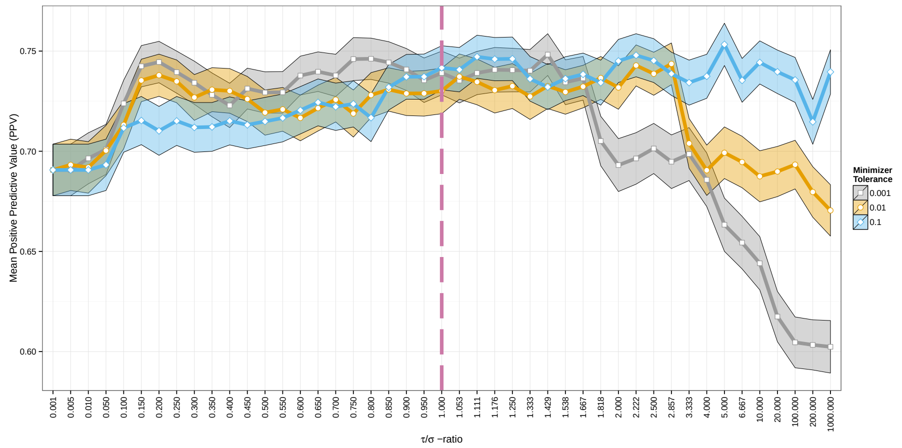

Figure 2: Parameter space evaluation for the method of Washietl *et al.* (2012). Plotted are the mean positive predictive values (PPV) for the entire benchmark data set using different parameter settings. For the sake of clarity, only three different values for the minimizer tolerance ϵ_m, namely 0.1, 0.01, and 0.001, are depicted, while for each of them a large range of τ/σ -ratios is used. The polygon surrounding each line of mean values indicates the standard deviation of PPVs within the entire set of predictions for the corresponding parameter setting. The dashed, purple, vertical line highlights the τ/σ -ratio used as default value for `RNApvmin`.

The benchmark results for all three methods that correspond to their default parameters are listed in Table 1. Estimation of the distribution of PPVs are shown in terms of 95% confidence intervals that we derive from a bootstrapping analysis with 1000 iterations, see Fig. 4.

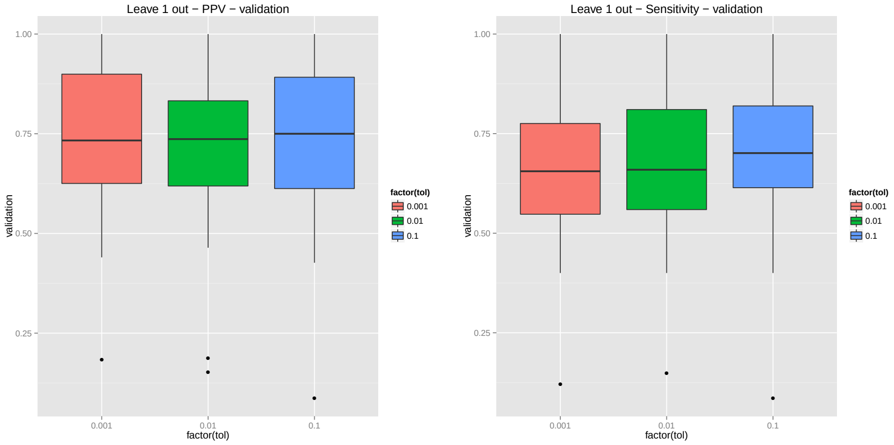

Figure 3: Leave-one-out analysis for the method of Washietl *et al.* (2012). Shown are Positive Predictive Values (PPV), left plot, and Sensitivity (TPR), right plot. Mean PPV for the three different tested minimizer tolerance of 0.001, 0.01, and 0.1 are 0.726, 0.700, and 0, 739 with standard deviations of 0.209, 0.226, and 0.216, respectively. The individual PPVs have been weighted by their contribution of predicted base pairs to the total number of predicted pairs in the entire data set.

Sequence	Length	Sensitivity				Positive Predictive Value				Pairing Probability Score				Ensemble Diversity				Ensemble Distance			
		RNAfold	Deigan	Zarringhalam	Washietl	RNAfold	Deigan	Zarringhalam	Washietl	RNAfold	Deigan	Zarringhalam	Washietl	RNAfold	Deigan	Zarringhalam	Washietl	RNAfold	Deigan	Zarringhalam	Washietl
Pre-Q1 riboswitch, B. subtilis*	34	0.63	0.63	0.63	0.63	1	1	1	1	0.6	0.59	0.56	0.59	0.03	0.05	0.05	0.02	0.11	0.11	0.12	0.11
Telomerase pseudoknot, human*	47	0.4	0.4	0.4	0.4	0.75	0.75	0.75	0.75	0.41	0.39	0.4	0.46	0.05	0.04	0.04	0.1	0.23	0.24	0.22	0.21
Fluoride riboswitch, P. syringae*	66	0.56	0.69	0.69	0.63	0.64	0.85	0.85	0.67	0.47	0.65	0.63	0.56	0.17	0.09	0.09	0.1	0.24	0.13	0.14	0.19
Adenine riboswitch, V. vulnificus	71	1	1	1	1	1	1	1	1	0.87	0.99	0.95	0.78	0.14	0.04	0.05	0.11	0.08	0.02	0.04	0.14
tRNA(asp), yeast	75	0.52	0.52	0.52	0.52	0.48	0.48	0.52	0.48	0.54	0.58	0.54	0.61	0.11	0.1	0.06	0.12	0.27	0.25	0.26	0.22
tRNA(phe), E. coli	76	1	1	0.81	1	1	1	0.71	1	0.5	0.97	0.84	0.62	0.31	0.04	0.12	0.14	0.29	0.02	0.13	0.22
TPP riboswitch, E. coli	79	0.77	0.95	0.95	0.5	0.85	0.88	0.88	0.44	0.58	0.85	0.87	0.59	0.17	0.12	0.08	0.12	0.21	0.11	0.1	0.23
SARS corona virus pseudoknot*	82	0.69	0.69	0.69	0.69	0.86	0.86	0.86	0.86	0.44	0.68	0.69	0.55	0.24	0.07	0.03	0.1	0.34	0.16	0.13	0.25
cyclic-di-GMP riboswitch, V. cholerae	97	0.83	0.93	0.62	0.96	0.83	0.93	0.62	0.92	0.77	0.84	0.8	0.79	0.12	0.08	0.11	0.06	0.11	0.08	0.11	0.12
SAM I riboswitch, T. tengcongensis*	118	0.79	0.82	0.77	0.79	0.91	0.97	0.97	0.97	0.78	0.78	0.77	0.73	0.09	0.07	0.05	0.05	0.11	0.11	0.09	0.14
5S rRNA, E. coli	120	0.29	0.97	0.83	0.86	0.24	0.92	0.74	0.75	0.28	0.87	0.67	0.56	0.2	0.1	0.21	0.16	0.46	0.1	0.18	0.28
M-Box riboswitch, B. subtilis	154	0.88	0.88	0.88	0.88	0.91	0.91	0.93	0.91	0.84	0.85	0.84	0.82	0.07	0.04	0.04	0.05	0.09	0.08	0.07	0.1
P546 domain, bI3 group I intron	155	0.64	0.98	0.68	0.8	0.75	0.98	0.86	0.9	0.65	0.92	0.64	0.61	0.25	0.08	0.11	0.12	0.23	0.06	0.18	0.23
Lysine riboswitch, T. maritime*	174	0.79	0.7	0.7	0.56	0.89	0.79	0.83	0.64	0.78	0.73	0.75	0.7	0.09	0.08	0.09	0.08	0.12	0.15	0.12	0.17
Group I intron, Azoarcus sp.*	214	0.6	0.71	0.63	0.54	0.61	0.78	0.82	0.57	0.52	0.69	0.69	0.54	0.21	0.1	0.08	0.14	0.28	0.16	0.14	0.25
Signal recognition particle RNA, human	301	0.11	0.58	0.14	0.27	0.12	0.58	0.16	0.28	0.1	0.54	0.25	0.32	0.17	0.08	0.18	0.18	0.59	0.3	0.46	0.44
Hepatitis C virus IRES domain*	336	0.31	0.81	0.78	0.75	0.29	0.87	0.86	0.86	0.31	0.8	0.75	0.48	0.17	0.03	0.06	0.17	0.43	0.1	0.12	0.3
RNase P, B. subtilis*	401	0.56	0.73	0.7	0.7	0.52	0.75	0.79	0.73	0.59	0.69	0.72	0.54	0.13	0.09	0.09	0.14	0.25	0.17	0.14	0.26
Group II intron, O. iheyensis*	412	0.89	0.67	0.69	0.81	0.97	0.77	0.83	0.89	0.61	0.64	0.65	0.5	0.27	0.08	0.13	0.16	0.22	0.2	0.17	0.29
Group I Intron, T. thermophila*	425	0.71	0.84	0.77	0.68	0.66	0.84	0.79	0.65	0.73	0.82	0.77	0.71	0.15	0.1	0.09	0.1	0.18	0.11	0.13	0.18
5' domain of 16S rRNA, H. volcanii	473	0.88	0.88	0.86	0.87	0.79	0.81	0.82	0.81	0.83	0.86	0.85	0.79	0.11	0.05	0.05	0.07	0.13	0.11	0.11	0.15
HIV-1 5' pseudoknot domain*	500	0.34	0.49	0.51	0.43	0.34	0.5	0.6	0.45	0.36	0.47	0.45	0.39	0.22	0.11	0.15	0.17	0.38	0.31	0.29	0.35
5' domain of 23S rRNA, E. coli	511	0.78	0.85	0.87	0.62	0.61	0.71	0.74	0.52	0.76	0.84	0.84	0.72	0.17	0.1	0.11	0.11	0.17	0.13	0.12	0.18
5' domain of 16S rRNA, E. coli	530	0.63	0.89	0.81	0.75	0.54	0.79	0.79	0.69	0.61	0.87	0.8	0.7	0.14	0.05	0.07	0.1	0.26	0.11	0.13	0.19
Mean		0.65	0.78	0.71	0.69	0.69	0.82	0.78	0.74	0.58	0.75	0.70	0.61	0.16	0.07	0.09	0.11	0.24	0.14	0.15	0.22
SD		0.23	0.17	0.18	0.19	0.26	0.15	0.18	0.20	0.20	0.16	0.16	0.13	0.07	0.03	0.05	0.04	0.13	0.08	0.09	0.08

Table 1: Performance measures for our benchmark dataset. The table shows *Sensitivity*, *Positive Predictive Value*, as well as the *Pairing Probability Score*, and the normalized *Ensemble Diversity* and distance between ensemble and reference structure for the three distinct guided secondary structure prediction approaches. The values are normalized by dividing by the sequence length n. For comparison to the unguided prediction we use `RNAfold`. The performance values for the Washietl *et al.* (2012) method are averages over 16 individual runs of the optimization.

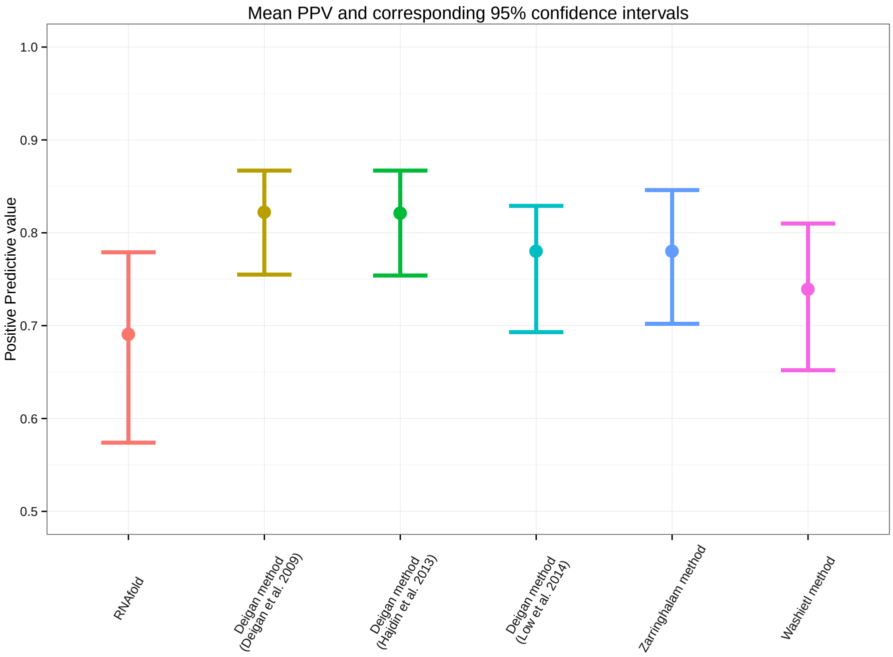

Figure 4: Confidence interval estimation for the Positive Predictive Values (PPV). To assess the distribution of the performance benchmarks sample mean, we performed a bootstrapping analysis using 1000 samples. From this resampling, we derive 95% confidence intervals of the mean PPV. For comparison we added performance benchmark results for three different parameter sets of the Deigan *et al.* (2009) method, ($m = 2.6$ $b = -0.8$), ($m = 1.8$, $b = -0.6$), and ($m = 3.0$, $b = -0.6$), taken from the original publication Deigan *et al.* (2009), from Hajdin *et al.* (2013), and Low *et al.* (2014), respectively. The second parameter set corresponds to the default parameters we use in our implementation. Although there is a large overlap between the different methods, the approach of Deigan *et al.* (2009) shows the best average performance on our benchmark set.

6 Theophylline sensing riboswitch

As an additional, particular example for comparing the three methods we investigated the artificially designed RNA switch *theo-P-is10* described by Qi *et al.* (2012). This RNA consists of a theophylline sensing aptamer part followed by a ncRNA expression platform. The switching principle follows a regular ON-switch behavior, where under sufficiently high concentrations of theophylline, the aptamer part of the structure is thermodynamically favored, and the downstream located ncRNA part of the sequence folds into its active state. On the other hand, in the absence of theophylline, the expression platform misfolds into an inactive state.

In the original design *theo-P-is10* forms a pseudoknot interaction between the aptamer stem and the expression platform in the inactive state. However, by

using `RNAfold` we explicitly exclude pseudoknots, which is also true for almost all other secondary structure prediction programs available. Nevertheless, the data that comes with the work of Qi *et al.* (2012) provides a rich source of interesting SHAPE probing data, since it consists of normalized reactivities from two experiments: (i) the RNA folds in theophylline free solution, and (ii) the RNA folds in the presence of 0.5 mM theophylline, respectively. Since the designed pseudoknot is only present in the inactive state of the RNA switch, i.e. in theophylline-free solution, we do not emphasize too much on the correctness of the structure prediction in this case.

To compare the different variants of guided RNA secondary structure prediction through SHAPE reactivity data incorporation for *theo-P-is10*, we computed the ground state structures, and base pair probabilities for the two corresponding experimental data sets. Instead of using two dotplots for comparison, we create differential `RNAbow` plots (Aalberts and Jannen, 2013) to visualize the difference in base pair probability predictions. Here, a differential `RNAbow` plot consists of two sets of arcs located on the upper and lower half of the horizontally aligned nucleotide sequence, showing the predictions for both experiments, respectively. The strength/width of the arcs represents the pairing probability (thicker lines mean higher probability), whereas arcs are colored with an intensity corresponding to the absolute value of difference in predictions (red in the upper half, blue in the lower half) only, if pairing probability is higher when compared to the other experiment. Otherwise, arcs are drawn in gray, indicating lower probability compared to the other experiment. For better visualization we restrict the `RNAbow` plots to pairing probabilities of 0.1 and above.

Figure 5 outlines the two ground state structures of the designed RNA switch together with their corresponding pairing probabilities in form of a bowplot. Results of the predictions using the method of Deigan *et al.* (2009) with default parameters, the parameters used in Qi *et al.* (2012), the method of Zarringhalam *et al.* (2012) with default parameters, and the method of Washietl *et al.* (2012), are shown in Figures 6, 7, 8, and 9, respectively. It can be easily seen that the method of Deigan *et al.* (2009) using default parameters clearly misses the proposed ground state structures and essentially yields results as obtained by `RNAfold` without incorporation of SHAPE reactivities. On the other hand, using the two parameters $m = 3.4$, and $b = -0.5$, both SHAPE reactivity data sets yield high probabilities for the aptamer pocket and the functional ncRNA part, although only in presence of theophylline the aptamer pocket is fully formed. Using the method of Zarringhalam *et al.* (2012) both predicted ground state structures again correspond to the active conformation of the designed RNA switch. However, the proposed pseudoknot interaction between the two hairpin loops of the inactive state becomes visible in the base pair plot. This effect is even more pronounced when using the method of Washietl *et al.* (2012). Here, the pairing probabilities for the pseudoknot interaction are much higher in the absence of theophylline, whereas the probabilities of the base pairs involved in the formation of the aptamer pocket and the ncRNA part are increased in the presence of theophylline. Nevertheless, both ground state structures are virtually identical and represent the active conformation.

In contrast to the above methods, the implementation of so-called *soft-constraints* in the ViennaRNA Package 2.2 (published elsewhere) also allows for a direct inclusion of binding free energies of the ligand to the aptamer pocket. For this purpose, the ensemble of structures is modified such that all structures

that exhibit the aptamer pocket receive an additional stabilizing free energy of $E_s = -9.22$ kcal/mol, according to the dissociation constant of $K_d = 0.32\mu M$ taken from Jenison *et al.* (1994). The resulting constrained secondary structure prediction is shown in Figure 10. Here, the shift towards the functional ligand binding state of the RNA switch under presence of theophylline is clearly visible in the base pair probabilities.

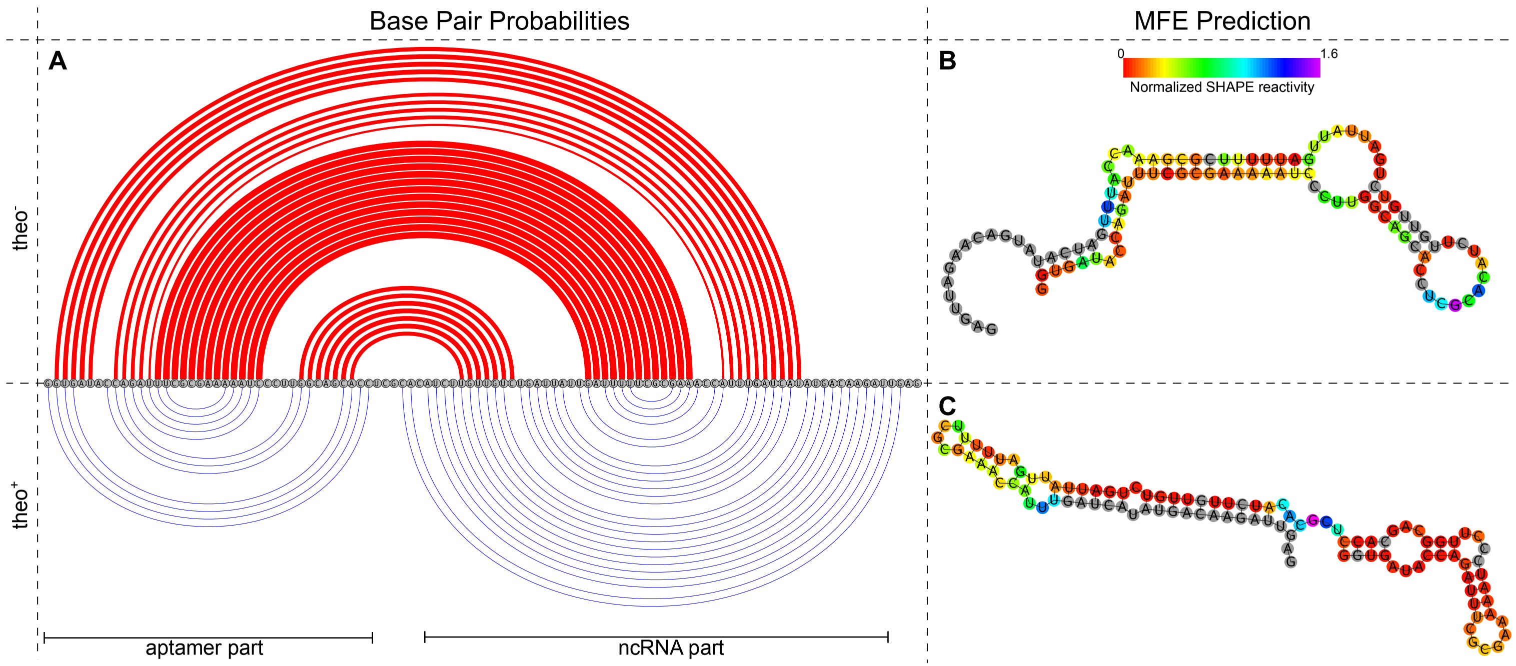

Figure 5: The model system of the designed theophylline Riboswitch (Qi *et al.*, 2012).
A Base pair probabilities of the two proposed secondary structure states as computed by `RNAfold` depicted by an `RNAbow` diagram. Similar to the differential `RNAbow` plot, base pair probabilities are depicted by the strength/width of the arcs. However, the upper half (red arcs) shows the base pairs of the MFE structure (supposedly OFF state, since the actual conformation has not been validated experimentally), and the lower half (blue arcs) the ON-state, respectively, instead of the difference between two distinct predictions. Secondary structure drawings of the MFE structure and the ON-state with a color encoding of their respective SHAPE reactivity as measured by Qi *et al.* (2012) are shown in **B**, and **C**, respectively.

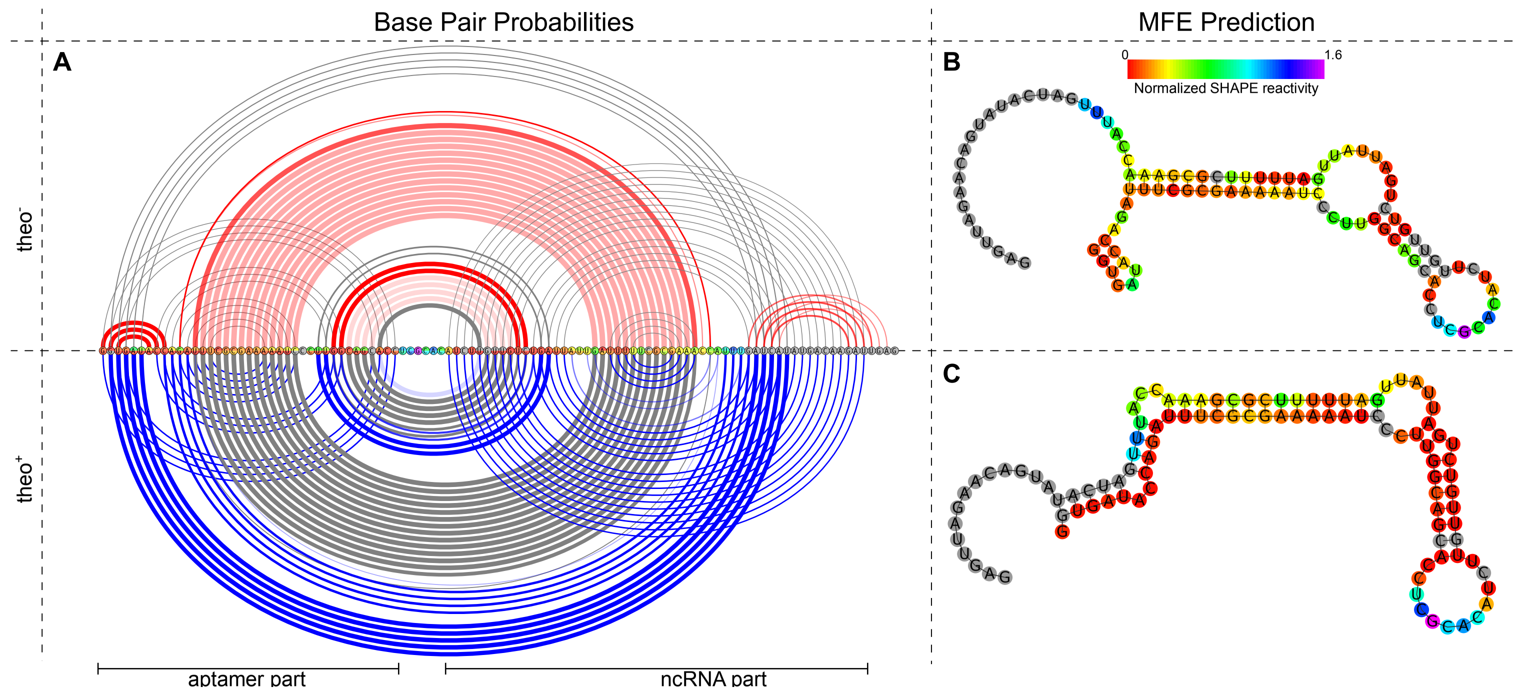

Figure 6: Guided secondary structure prediction with `RNAfold` using the method of Deigan *et al.* (2009) with default parameters ($m = 1.8$ and $b = -0.6$).

A Differential `RNAbow` diagram of the base pair probabilities (see text). SHAPE reactivity data for the theophylline-free probing experiment (upper half), and a concentration of 0.5 mM theophylline (lower half) are taken from Qi *et al.* (2012). Although the (short-range) aptamer part and ncRNA part of *theo-P-is10* show relatively high pairing probabilities, especially in the presence of theophylline (lower half, blue arcs), long-range interactions dominate the structure ensemble in both cases.

The two secondary structure plots **B**, and **C** show the predicted MFE structures for the theophylline-free system, and under presence of theophylline, respectively. Colored nucleotides show the corresponding SHAPE reactivity data, whereas grey circles indicate the absence of probing data.

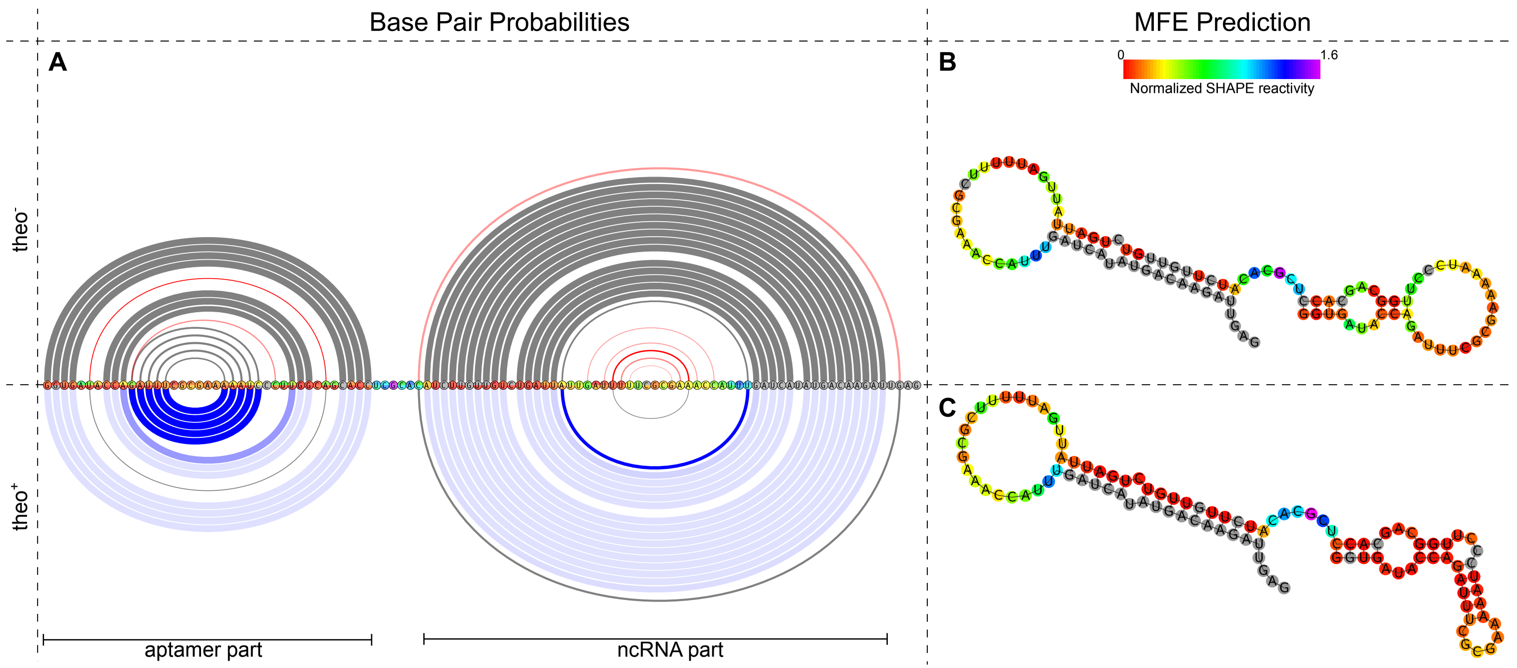

Figure 7: Guided secondary structure prediction with `RNAfold` using the method of Deigan *et al.* (2009) with parameters taken from Qi *et al.* (2012) ($m = 3.4$ and $b = -0.5$).

A Differential `RNAbow` diagram of the base pair probabilities. SHAPE reactivity data for the theophylline-free probing experiment (upper half), and a concentration of 0.5 mM theophylline (lower half) are taken from Qi *et al.* (2012). Here, both predictions show a shift of the structure ensemble towards the functional ON-state of the RNA switch. Except for the innermost stem of the aptamer part of the designed RNA, pairing probabilities of the (dominant) functional state are only slightly higher in the presence of theophylline (light blue). The two secondary structure plots **B**, and **C** show the predicted MFE structures for the theophylline-free system, and under presence of theophylline, respectively. Colored nucleotides show the corresponding SHAPE reactivity data, whereas grey circles indicate the absence of probing data.

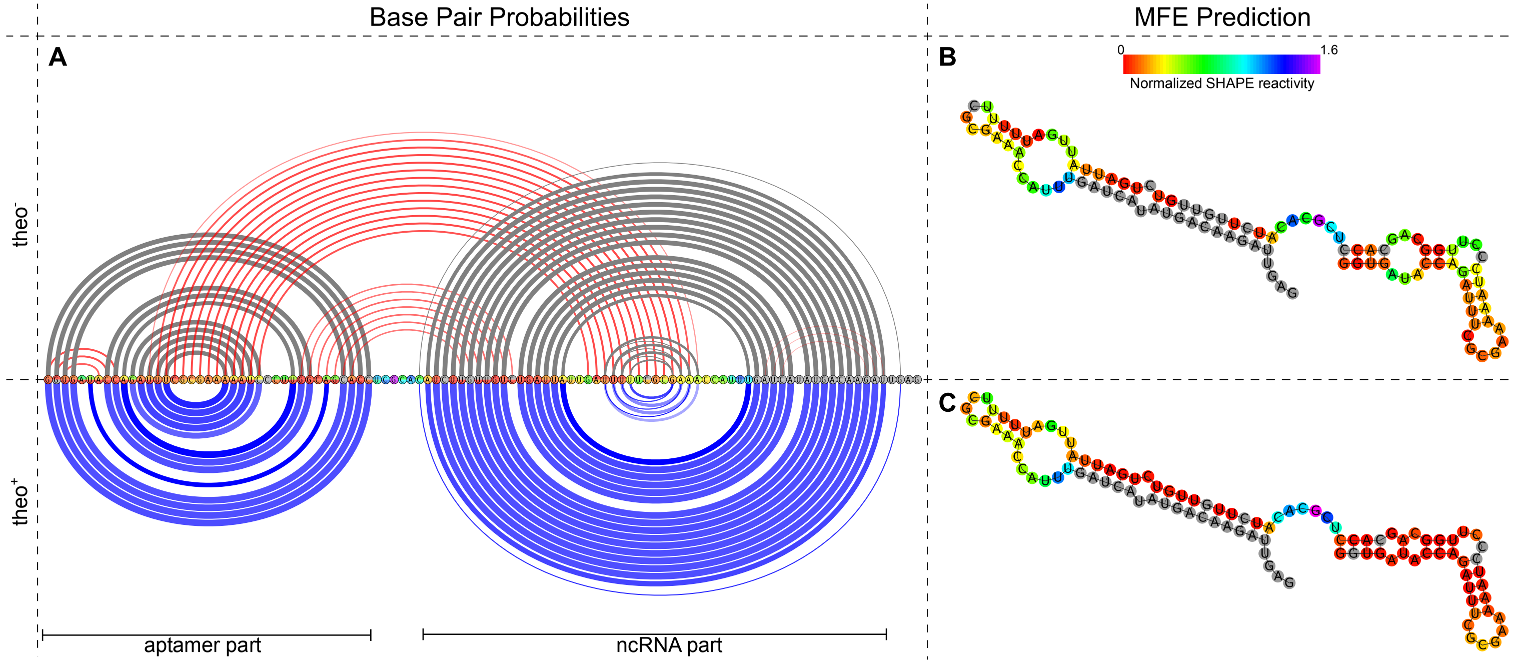

Figure 8: Guided secondary structure prediction with `RNAfold` using the method of Zarringhalam *et al.* (2012) with default parameters. **A** Differential `RNAbow` diagram of the computed base pair probabilities. SHAPE reactivity data for the theophylline-free probing experiment (upper half), and a concentration of 0.5 mM theophylline (lower half) are taken from Qi *et al.* (2012). Both data sets result in high probabilities for the aptamer, and ncRNA part. However, in the presence of theophylline, the possible long-range interaction between the two complementary hairpin loop sequences of these parts (upper half, large red arcs) falls below the probability threshold of 0.1 used for the construction of the `RNAbow` plot. This goes along with a high shift of the ensemble towards the ON-state of the riboswitch (lower half, blue arcs).

The two secondary structure plots **B**, and **C** show the predicted MFE structures for the theophylline-free system, and under presence of theophylline, respectively. Colored nucleotides show the corresponding SHAPE reactivity data, whereas grey circles indicate the absence of probing data. Both SHAPE reactivity data sets result in a MFE prediction of the ON-state, with the only difference of an additional A-U base pair in the ligand binding pocket of the aptamer part under the presence of theophylline.

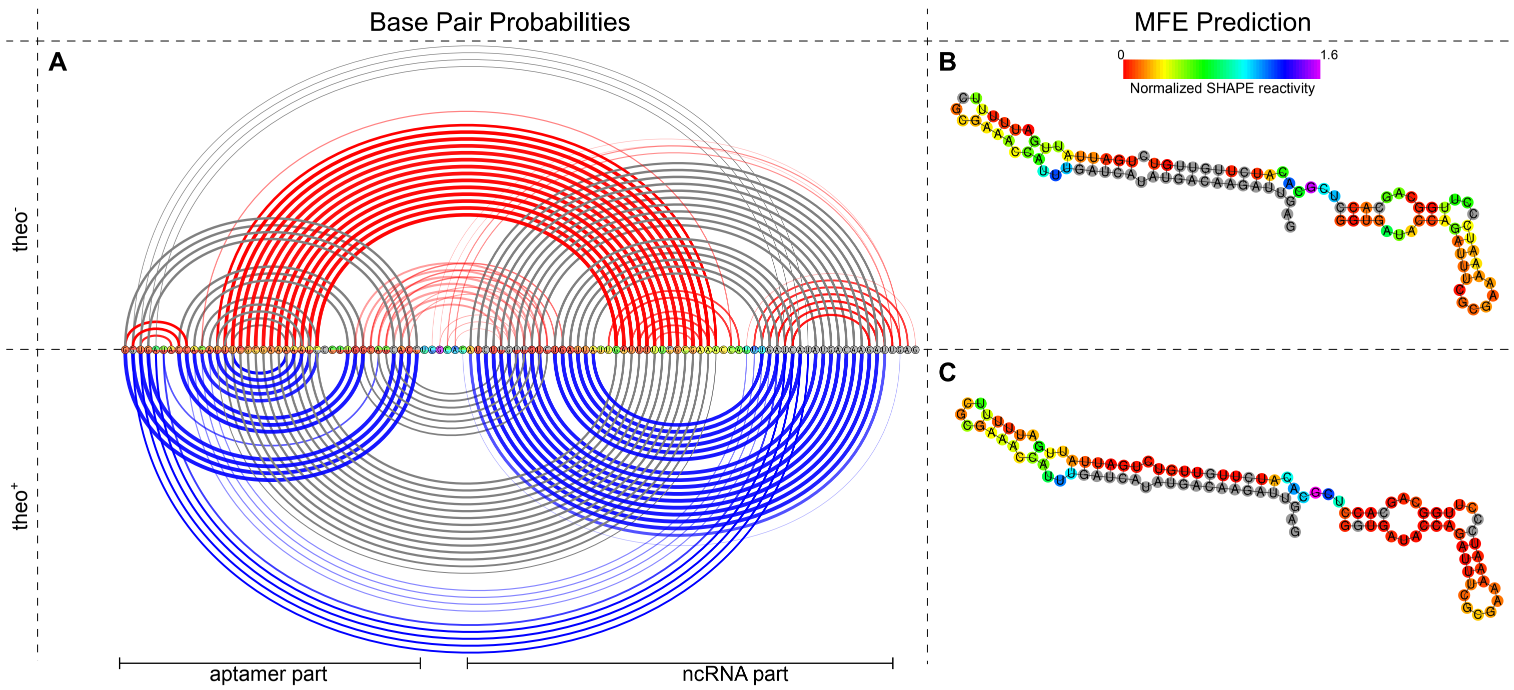

Figure 9: Guided secondary structure prediction with `RNAfold` using the method of Washietl *et al.* (2012) with default parameters. Here, the two provided SHAPE reactivity data sets were used to compute a position-wise perturbation vector for the usage in guided secondary structure prediction. **A** Differential `RNAbow` diagram of the computed base pair probabilities. SHAPE reactivity data for the theophylline-free probing experiment (upper half), and a concentration of 0.5 mM theophylline (lower half) are taken from Qi *et al.* (2012). The two different modes (OFF/ON state) are clearly visible in the differential probabilities. While under theophylline-free condition (upper half) a long range interaction between the two complementary hairpin loop parts of the aptamer and the ncRNA show rather high probability (red arcs), this is not the case under the presence of theophylline (lower half). Here, the base pairs of the aptamer part as well as the ncRNA part of the RNA switch exhibit distinctly high pairing probability (dark blue arcs). The two secondary structure plots **B**, and **C** show the predicted MFE structures for the theophylline-free system, and under presence of theophylline, respectively. Colored nucleotides show the corresponding SHAPE reactivity data, whereas grey circles indicate the absence of probing data. Although the pairing probabilities as shown in **A** would suggest otherwise, both predicted ground state structures represent the ON-state of the RNA switch.

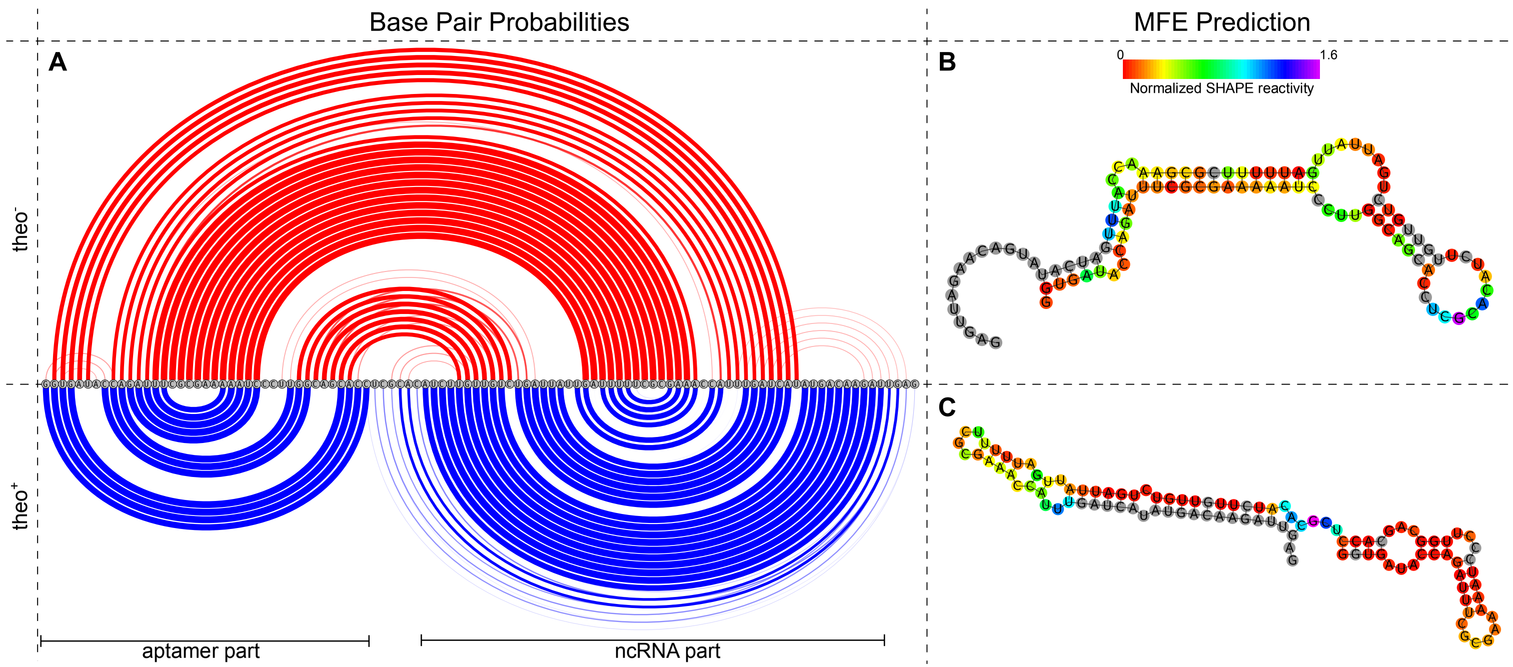

Figure 10: Direct incorporation of theophylline binding free energy using the *soft constraints* feature of the ViennaRNA Package 2.2. Here, structures in the lower part of the figure ($theo^+$) receive an additional stabilizing free energy of $E_s = -9.22$ kcal/mol if they exhibit the aptamer pocket. This soft constraint is derived from the dissociation constant $K_d = 0.32\mu M$ taken from Jenison *et al.* (1994) -9.22. The upper part ($theo^-$) consists of data obtained by `RNAfold` with default parameters. **A** Differential `RNAbow` diagram of the computed base pair probabilities. The two secondary structure plots **B**, and **C** show the predicted MFE structures for the theophylline-free system, and under presence of theophylline, respectively. For comparison with the corresponding experimentally measured SHAPE reactivity data, nucleotides are colored according to this data. Grey circles indicate the absence of probing data. The ON and OFF state of the RNA switch are clearly visible in both, the ground state and the base pair probability predictions.

SHAPE reactivity	Probability for being unpaired
<0.25	0.00 - 0.35
0.25 - 0.30	0.35 - 0.55
0.30 - 0.70	0.55 - 0.85
>0.70	0.85 - 1.00

Table 2: Linear mapping classes used to convert SHAPE reactivities to probabilities for being unpaired according to Zarringhalam *et al.* (2012).

7 Mapping SHAPE reactivities to pairing probabilities

While the approach of Deigan *et al.* (2009) directly converts SHAPE reactivities to pseudo energies, the methods of Zarringhalam *et al.* (2012) and Washietl *et al.* (2012) both require experimentally determined pairing probabilities as input data. However, converting raw reactivity values to pairing probabilities is not a trivial task and both approaches use different methods to calculate pairing probabilities based on given SHAPE reactivities. While Washietl et al. used a simple cutoff approach to distinguish between paired and unpaired positions, Zarringhalam et al. used a more sophisticated method where the normalization is carried out in a stepwise linear fashion (See table 2).

In this benchmark a common method was used to compute the required pairing probabilities based on the experimentally determined SHAPE reactivities. The application `RNAplfold` was used to predict the pairing probabilities for all sequences of the benchmark. The predicted pairing probabilities of all nucleotides were then compared with the determined SHAPE reactivities. The dataset containing about 4500 observations showed a significant correlation between the logarithm of the SHAPE reactivity and the probability for a certain nucleotide to be unpaired. However, as shown in figure 11, the experimental signal shows a high variation and high reactivities can also be observed for paired nucleotides, and *vice versa*. Nevertheless, a linear model is suitable for converting the logarithm of the SHAPE reactivity to the probability for being unpaired. The best fit is

$$q = \frac{5}{8}(2.29 + \log(\text{SHAPE reactivity})) \tag{5}$$

Since the equation above may also lead values of q below 0 or larger than 1, all results exceeding those limits are replaced by 0 or 1, respectively.

8 Running time

The impact of the incorporation of additional soft constraints onto the required amount of computational time was benchmarked for the whole dataset using a workstation (Intel Core 2 Quad 2.83 GHz, GCC 4.8.2). The running time for the folding recursion are reported as averages over 10 runs. As shown in figure 12, the incorporation of additional constraints results in a slight increase of the required computational time. However, the effect is less pronounced for the

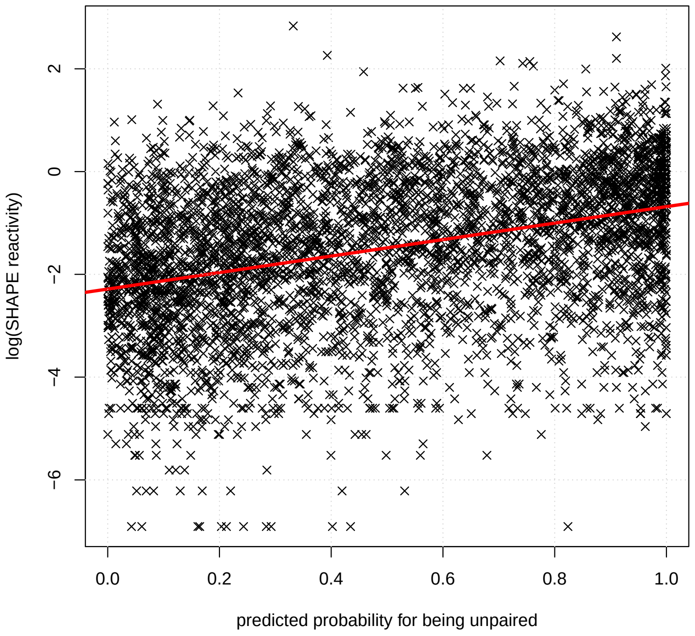

Figure 11: Relation of measured SHAPE reactivities to predicted probabilities for being unpaired

approach of Deigan et al., which may be explained by the fact that in contrast to the other approaches, which apply pseudo energies for every paired/unpaired nucleotide, the free energy is only adapted when evaluating stacked pairs.

The overall running time for the prediction according to Washietl et al. can be separated into two phases. First, a perturbation vector is calculated by numerically minimizing an objective function. This step requires most of the computational resources since the exact evaluation of the gradient at various points of the minimization algorithm scales at $O(N^4)$. However, the evaluation can be done much faster when the gradient is estimated from a number of sampled sequences. Second, the calculated perturbation vector is used to constrain the secondary structure prediction.

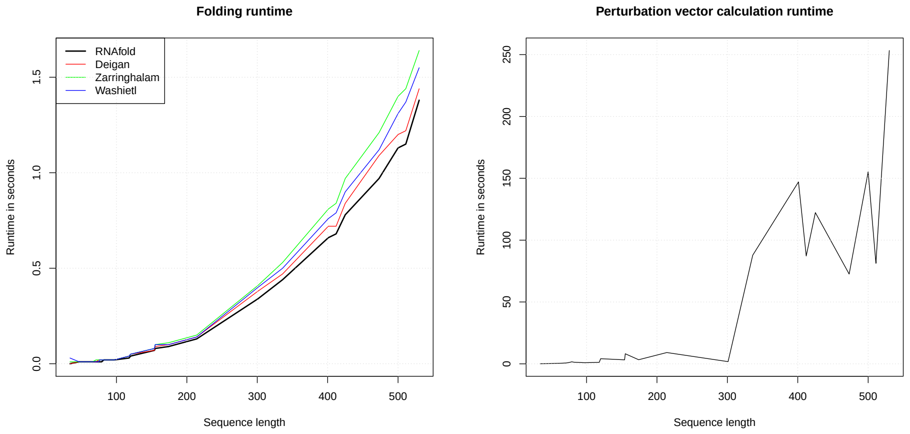

Figure 12: Running time for predicting the MFE structure and the partition function using various approaches and running time required to calculate a perturbation vector with exact gradient evaluation

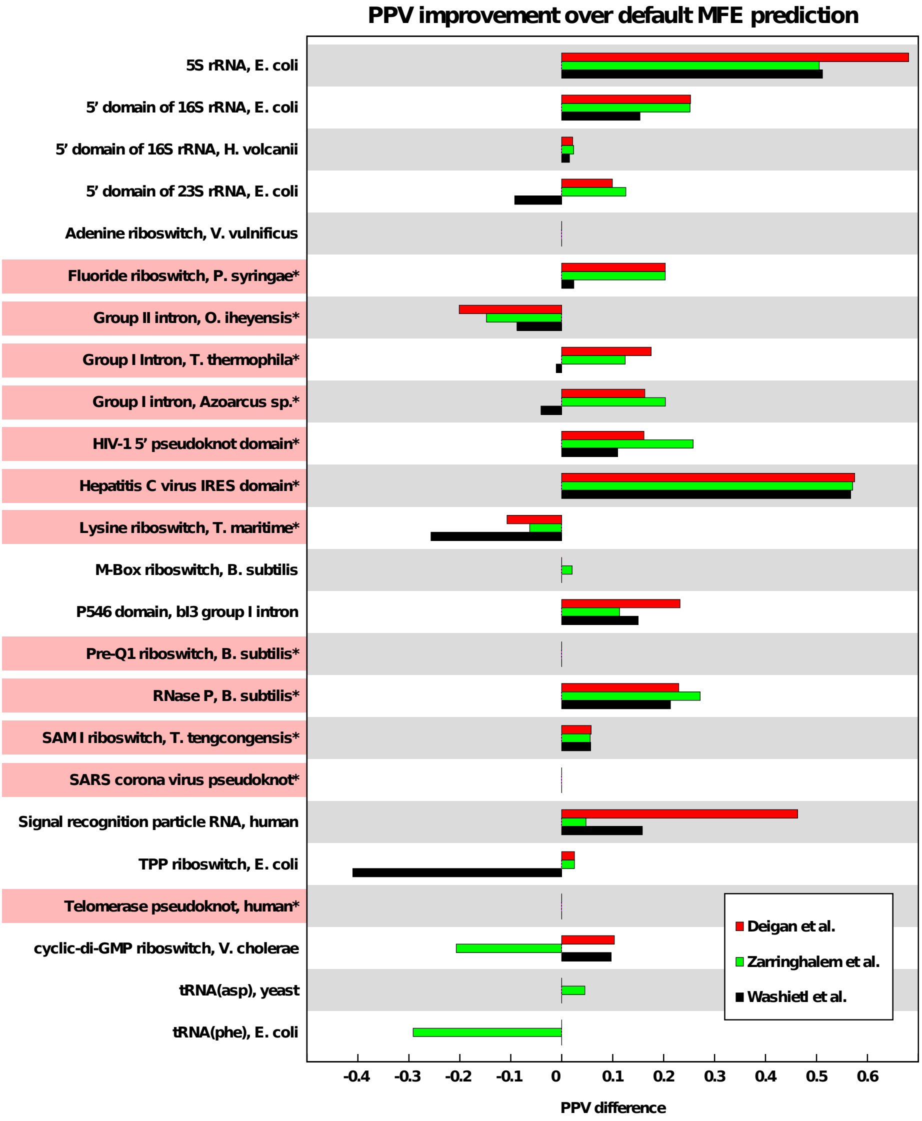

Figure 13: Change of the PPV of the minimum free energy structure due to constrained folding compared to unconstrained folding. Reference structures that contain pseudoknots are marked by an asterisk and light-red background. The poor performance of the Washietl *et al.* (2012) method in the case of *E.coli TPP riboswitch* is caused by an inconsistency between SHAPE reactivity and proposed reference structure.

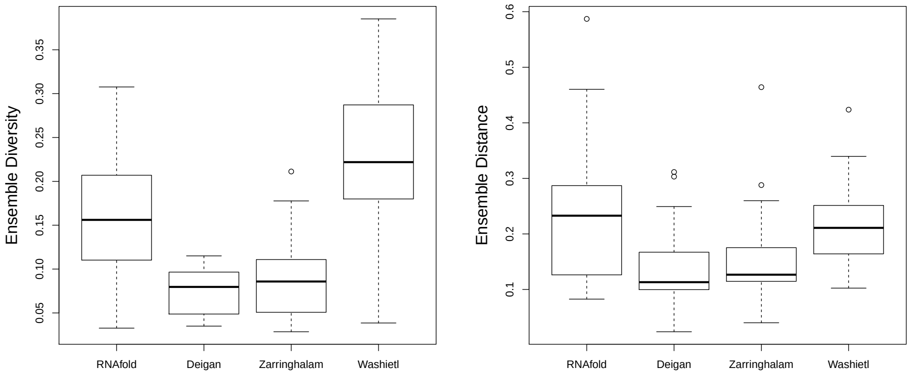

Figure 14: *Ensemble Diversity* and *Ensemble distance* to the reference structure for unconstrained and constrained structure predictions.

References

Aalberts, D. P. and Jannen, W. K. (2013). Visualizing RNA base-pairing probabilities with RNAbow diagrams. *RNA*, **19**(4), 475–478.

Bernhart, S. H., Hofacker, I. L., Will, S., Gruber, A. R., and Stadler, P. F. (2008). `RNAalifold`: improved consensus structure prediction for RNA alignments. *BMC Bioinformatics*, **9**, 474.

Cordero, P., Kladwang, W., VanLang, C. C., and Das, R. (2012). Quantitative dimethyl sulfate mapping for automated RNA secondary structure inference. *Biochemistry*, **51**, 7037–7039.

Deigan, K. E., Li, T. W., Mathews, D. H., and Weeks, K. M. (2009). Accurate SHAPE-directed RNA structure determination. *PNAS*, **106**, 97–102.

Eddy, S. R. (2014). Computational analysis of conserved RNA secondary structure in transcriptomes and genomes. *Annu Rev Biophys*, **43**, 433–456.

Hajdin, C. E., Bellaousov, S., Huggins, W., Leonard, C. W., Mathews, D. H., and Weeks, K. M. (2013). Accurate SHAPE-directed RNA secondary structure modeling, including pseudoknots. *Proceedings of the National Academy of Sciences*, **110**(14), 5498–5503.

Harmanci, A. O., Sharma, G., and Mathews, D. H. (2011). TurboFold: Iterative probabilistic estimation of secondary structures for multiple RNA sequences. *BMC Bioinformatics*, **12**, 108.

Hofacker, I. L., Fontana, W., Stadler, P. F., Bonhoeffer, L. S., Tacker, M., and Schuster, P. (1994). Fast folding and comparison of RNA secondary structures. *Chemical Monthly*, **125**, 167–188.

Hofacker, I. L., Fekete, M., and Stadler, P. F. (2002). Secondary structure prediction for aligned RNA sequences. *J. Mol. Biol.*, **319**, 1059–1066.

Jenison, R. D., Gill, S. C., Pardi, A., and Polisky, B. (1994). High-resolution molecular discrimination by RNA. *Science*, **263**(5152), 1425–1429.

Kertesz, M., Wan, Y., Mazor, E., Rinn, J. L., Nutter, R. C., Chang, H. Y., and Segal, E. (2010). Genome-wide measurement of RNA secondary structure in yeast. *Nature*, **467**, 103–107.

Low, J. T., Garcia-Miranda, P., Mouzakis, K. D., Gorelick, R. J., Butcher, S. E., and Weeks, K. M. (2014). Structure and dynamics of the HIV-1 frameshift element RNA. *Biochemistry*, **53**(26), 4282–4291.

Mathews, D. H., Sabina, J., Zuker, M., and Turner, D. H. (1999). Expanded sequence dependence of thermodynamic parameters improves prediction of RNA secondary structure. *J Mol Biol*, **288**, 911–940.

Mathews, D. H., Disney, M. D., Childs, J. L., Schroeder, S. J., Zuker, M., and Turner, D. H. (2004). Incorporating chemical modification constraints into a dynamic programming algorithm for prediction of RNA secondary structure. *Proc. Natl. Acad. Sci. USA*, **101**, 7287–7292.

Qi, L., Lucks, J. B., Liu, C. C., Mutalik, V. K., and Arkin, A. P. (2012). Engineering naturally occurring trans-acting non-coding RNAs to sense molecular signals. *Nucleic acids research*, **40**(12), 5775–5786.

Wan, Y., Qu, K., Zhang, Q. C., Flynn, R. A., Manor, O., Ouyang, Z., Zhang, J., Spitale, R. C., Snyder, M. P., Segal, E., and Chang, H. Y. (2014). Landscape and variation of RNA secondary structure across the human transcriptome. *Nature*, **505**, 706–709.

Washietl, S., Hofacker, I. L., Stadler, P. F., and Kellis, M. (2012). RNA folding with soft constraints: reconciliation of probing data and thermodynamics secondary structure prediction. *Nucleic Acids Research*, **40**(10), 4261–4272.

Zarringhalam, K., Meyer, M. M., Dotu, I., Chuang, J. H., and Clote, P. (2012). Integrating chemical footprinting data into RNA secondary structure prediction. *PLOS ONE*, **7**(10).

Zuker, M., Jaeger, J. A., and Turner, D. H. (1991). A comparison of optimal and suboptimal RNA secondary structures predicted by free energy minimization with structures determined by phylogenetic comparison. *Nucleic Acids Res.*, **19**, 2707–2714.
